# Application of Pt@ZIF-8 nanocomposite-based electrochemical biosensor for sensitive diagnosis of tau protein in Alzheimer’s disease patients

**DOI:** 10.1038/s41598-023-43180-0

**Published:** 2023-09-27

**Authors:** Forough Chakari-Khiavi, Arezoo Mirzaie, Balal Khalilzadeh, Hadi Yousefi, Rozita Abolhasan, Amin Kamrani, Ramin Pourakbari, Koorosh Shahpasand, Mehdi Yousefi, Mohammad-Reza Rashidi

**Affiliations:** 1 Department of Medicinal Chemistry, Faculty of Pharmacy, Tabriz University of Medical Sciences, PO Box: 6446-14155, Tabriz, Iran; 2 Student Research Committee, Tabriz University of Medical Sciences, Tabriz, Iran; 3 Pharmaceutical Analysis Research Center, Tabriz University of Medical Sciences, Tabriz, Iran; 4 Stem Cell Research Center (SCRC), Tabriz University of Medical Sciences, Tabriz, 51664-14766 Iran; 5 Hematology and Oncology Research Center, Tabriz University of Medical Sciences, Tabriz, Iran; 6 Department of Basic Medical Sciences, Khoy University of Medical Sciences, Khoy, Iran; 7 Department of Immunology, Faculty of Medical Sciences, Tabriz University of Medical Sciences, Tabriz, Iran; 8 Department of Stem Cells and Developmental Biology, Cell Science Research Center, Royan Institute for Stem Cell Biology and Technology, Academic Center for Education, Culture and Research (ACECR), Tehran, 1665659911 Iran; 9 Research Center for Pharmaceutical Nanotechnology (RCPN), Tabriz University of Medical Sciences, Tabriz, Iran

**Keywords:** Neurological disorders, Biomarkers, Neurology, Analytical chemistry, Electrochemistry

## Abstract

Alzheimer’s disease (AD) is a progressive brain disorder characterized by the ongoing decline of brain functions. Studies have revealed the detrimental effects of hyperphosphorylated tau (p-tau) protein fibrils in AD pathogenesis, highlighting the importance of this factor in the early-stage detection of AD conditions. We designed an electrochemical immunosensor for quantitative detection of the *cis* conformation of the p-tau protein (*cis*-p-tau) employing platinum nanoparticles (Pt NPs) supported on zeolitic imidazolate frameworks (ZIF) for modifying the glassy carbon electrode (GCE) surface. Under optimum conditions, the immunosensor selectively and sensitively detected cis-p-tau within the broad linear range of 1 fg mL^−1^ to 10 ng mL^−1^ and the low limit of detection (LOD) of 1 fg mL^−1^ with desired reproducibility and stability. Furthermore, the fabricated immunosensor's performance was examined for the cis-p-tau analysis in the serum of AD patients, indicating its accuracy and feasibility for real-sample analysis. Notably, this is the first application of Pt@ZIF-8 nanocomposite in fabricating a valid immunosensor for selective cis-p-tau detection, even in the presence of trans-p-tau. It is worth mentioning that the enzyme-linked immunosorbent assay (ELISA) reference technique is not able to evaluate pico- or femtomolar concentrations of cis-p-tau, making the fabricated immunosensor superior for early-stage measurement and screening of AD.

## Introduction

Alzheimer’s disease (AD) was first described in 1906 by Bavarian clinical psychiatrist and neuropsychologist Alois Alzheimer as an outlandishly intense disease occurring in the cerebral cortex. They reported a woman with developed dementia in her early 50s^1,2^. AD, a progressive neurodegenerative disease, is characterized by disorders in brain functions including remembering, and cognitive and behavioral abilities among the elderly^3,4^. According to WHO statistics, about 60–70% of dementia are because of AD^5^. Such a large population of AD cases inflicts irrefutable psychological and economic losses on human society^6^. Despite conducting a great deal of research on AD, the exact treatment has not been reported yet. In recent decades, all the proposed treatments have only slowed down the disease progression and relieved the symptoms^7^. It is noteworthy that the late onset manifestation of clinical symptoms in AD patients is one of the main reasons for the delay in starting drug therapy and the failure in treatment efficiency^8^. Great attempts have been devoted to developing diagnostic methods. Imaging techniques like computed tomography (CT)^9^, magnetic resonance imaging (MRI)^10^, positron emission tomography (PET), and single photon emission computed tomography (SPECT)^11^, along with enzyme-linked immunosorbent assay (ELISA)^12^, flexible multi-analyte profiling (xMAP)^13^, immunohistochemistry (IHC)^14^, and western blot^15^ are conventional methods for the diagnosis of AD. Despite all advantages of these techniques, numerous limitations have been reported in the clinical setting (Table 1)^16,17^.

**Table 1:** Conventional diagnostic methods of AD and their limitations.

Technique	Limitations
CT	Delivering large doses of X-ray radiation to body tissues
	Requiring expensive equipment and professional operators
	Costly and time-consuming for routine diagnosis
	Not accessible and affordable for all patients
MRI	Patient contraindications like motion artifacts and metal implants
	Time-consuming because of slow scanning velocity
	Requiring expensive equipment and professional operators
	Not accessible and affordable for all patients
PET	Not authorized as a stand-alone screening technique because of poor accuracy
	Time-consuming and laborious step of patient pre-preparation
	Requiring radiotracer agent for binding to target proteins (No radiotracer agent has been developed for tau protein detection)
	Requiring expensive equipment and professional operators
	Not accessible and affordable for all patients
xMAP	Probable cross-reactivity of antibodies
	Requiring optimization while developing new assays
	Providing results with low to medium resolution
	Not accessible and affordable for all patients
IHC	Inconstant and changeable antibody reactivity
	Relatively subjective data interpretation
	Non-specific background signal
	Variation depending on antibody selection, consolidation method, staining protocol
	Costly and time-consuming for routine diagnosis
	Not accessible and affordable for all patients
Western-Blot	Relatively medium-throughput
	Requiring high levels of protein lysate
	Results are greatly likely to any deviation in the case of any imbalance during the procedure
ELISA	Insensitive and time consuming
	Insensitive to low concentrations of targets
	Non-specific interactions
	False positives/negatives results
	Requiring large amounts of protein lysate

Thus, it is postulated that these techniques cannot be employed for wide, fast, routine diagnostics^18–20^. Today, the advent and development of Point-Of-Care (POC) techniques increase the efficiency of diagnosis along with the available methods. POC diagnostics provide considerably accurate, selective, and sensitive ease-of-use platforms^21–26^. Biosensors are promising bioanalysis instruments appropriate for POC detection of AD^19,27–31^. They can detect trace amounts of AD biomarkers in small volumes of biological samples in a short timeframe. Bioanalysis utilizing biosensors does not postulate costly benchtop instruments, spacious workplaces, challenging patient pre-preparation steps, or professionally trained operators^32–35^. Due to these advantages, biosensors are ideal candidates for real-time and on-site diagnosis of AD^34^.

Studies have brightened the extracellular aggregation of amyloid beta (Aβ) peptides and intraneuronal deposits of hyperphosphorylated tau (p-tau) protein as neuropathological characteristics of AD^36^. Since these pathological indications are likely to arise 10–15 years before the emergence of clinical symptoms, they are greatly apposite either for early-stage diagnosis of AD and its clinical course monitoring or drug development for AD cure^7^.

Tau protein was first figured out in 1975 by the Weingarten research team^37^. Tau is a subcategory of the microtubule-associated protein (MAP) family that plentifully occurs in the central nervous system (CNS)^38^. As the essential constituent of neurofibrillary tangles (NFT), tau plays a significant role in tubulin polymerization into microtubules (MTs) and conserving their stability^38^. Optimally, 3 mol of tau protein contain 2 mol of phosphate groups^37^. Abnormal posttranslational phosphorylation of tau leads to pathological conditions via two fundamental pathways: malfunction of the hyperphosphorylated tau protein in regulating MT assembly as well as the neurotoxic effect of hyperphosphorylated tau by intervening in normal tau’s biological role and generating tau aggregations^39,40^. Tau phosphorylation in serine (Ser) or threonine (Thr) residues before a proline (Pro) residue (pSer/Thr-Pro) engenders *cis* and *trans* conformational isoforms of tau protein with thoroughly different biological characteristics^41–43^. *Cis*-tau with low stability and poor function in MT assembly, tenacious potential for hyperphosphorylation, aggregation, and tangle formation strongly activates neurodegeneration mechanisms. Hence, it is frequently found in large concentrations in AD neurons. On the other hand, *trans*-tau is a distinguishing index of healthy neurons because of their innate faint tendency toward hyperphosphorylation, aggregation, and tangle formation, along with their potent nature for MT assembly and stability.

*Cis*-tau conformation is the earliest disclosed pathogenic indication of neurodegenerative diseases like Alzheimer’s^42,44^. Suggesting *cis*-tau as the specific biomarker for early diagnosis and treatment of AD, we put together the nanomaterials and electroimmunoassay to develop a biosensing platform. Applying nanomaterials improves the analytical performance of the biosensor owing to the high surface-to-volume ratio, conductivity, as well as catalytic activity^45–47^.

Metal‐organic frameworks (MOFs) have aroused much interest in the field of biosensing due to their high surface area and porosity^48,49^. Zeolitic imidazolate frameworks (ZIFs) are accounted for as a subfamily of MOFs with an extended three-dimensional structure made of tetrahedral metal cations linked by imidazolate (Im) anions^50,51^. Besides great mechanical and chemical stability, the porous structure with a narrow pore diameter endows a distinctive composition with a uniform distribution of NPs and fast electron transfer ability^52,53^. All these traits led us to apply Pt@ZIF-8 as an intuitive candidate for sensor surface modification.

Herein, we developed an electroimmunoassay in the light of nanoscale-sized zeolitic imidazolate frameworks (ZIFs). First, we synthesized and characterized the Pt@ZIF-8 nanocomposite. To fabricate the biosensor, the synthesized nanoparticle was electrochemically deposited on the glassy carbon electrode (GCE) surface employing the cyclic voltammetry (CV) technique. EDC/NHS was utilized to activate the carboxyl functional groups of the nanocomposite. Then, the specific antibody for *cis*-tau (anti-tau) was drop-casted on the sensing platform. Finally, we evaluated the analytical performance of the fabricated biosensor for *cis*-p-tau measurement in standard and serum samples. Our developed biosensor can detect *cis*-tau in the linear range of 1 fg mL^−1^–10 ng mL^−1^ which completely covers the physiological range of tau protein (∼ 300−600 pg mL^−1^). The analytical results approved the detection accuracy, selectivity, and sensitivity of the biosensing assay in an estimated short time.

## Methods

### Reagents and apparatus

Potassium ferro/ferricyanide K_3_/K_4_[Fe(CN)_6_] was bought from Aladdin Company (Shanghai, China). Polyethylene glycol 6000, 1-Ethyl-3-(3-dimethylaminopropyl) carbodiimide (EDC), and N-Hydroxysuccinimide (NHS) were purchased from Sigma-Aldrich (USA). Sulfuric acid 98% (H_2_SO_4_), ethanol (C_2_H_6_O), nitric acid (HNO_3_), disodium phosphate (Na_2_HPO_4_), monopotassium phosphate (KH_2_PO_4_), Zinc nitrate hexahydrate (Zn(NO_3_)_2_.6H_2_O), and citric acid (C_6_H_8_O_7_) were bought from Merck (Darmstadt, Germany). *Cis/Trans* p-tau was acquired from R&D (Minneapolis, MN, USA). Human serum samples were obtained from Shahid Ghazi Hospital, affiliated with Tabriz University of Medical Sciences (Tabriz, Iran). Distilled water was used for solution preparation and all the experimental tests.

All electrochemical tests, including electrochemical impedance spectroscopy (EIS), cyclic voltammetry (CV), and differential pulse voltammetry (DPV), were conducted in a conventional three-electrode system consisting of a platinum (Pt) wire as the counter electrode, Ag/AgCl (with 3 M KCl) as the reference electrode, and a 2-mm-diameter glassy carbon electrode (GCE) as the working electrode. This system was powered by an Autolab potentiostat/galvanostat (Metrohm) electrochemical system using NOVA 1.8 software for data analysis. The GCE was purchased from the Azar Company (Urmia, Iran). Successful surface modification of the GCE was investigated by FE-SEM (high-resolution field emission scanning electron microscope, VEGA TESCAN, Czech Republic), and the chemical constituents of the nanocomposite were surveyed by energy dispersive X-ray spectroscopy (EDX) coupled with the FE-SEM instrument.

### Synthesis of Pt@ZIF-8 nanocomposite

Pt@ZIF-8 nanocomposite was synthesized according to the straightforward one-step method reported previously by Li et al.^54^. First, colloidal Pt NPs were prepared. With this aim, H_2_PtCl_6_·6H_2_O was chemically reduced by ethylene glycol in the presence of sodium acetate as a stabilizing agent^55^. This reaction was conducted at 160 °C for 3h. Next, 12 mL of the prepared colloidal solution of Pt NPs and 11.35 g of 2‐methy imidazole were diffused in H_2_O. Then, 4 mL of the Zn(NO_3_)_2_·6H_2_O aqueous solution was put into the reaction mixture and stirred for 1.5 h at 25 °C. Finally, the solution was centrifuged, and black powder was collected, washed twice with H_2_O followed by methanol, and dried under vacuum at 120 °C for 12 h. 0.01 g of the synthesized Pt@ZIF-8 was ultrasonically dispersed in 1 ml of ultrapure distilled water for 2 h. Then, 600 µL of the obtained mixture was added to 4.4 ml of NaNO_3_ (0.1 M). The Pt@ZIF-8 solution was sonicated for 30 min before each electrodeposition step.

### Ethics approval and consent to participate

All AD cases were queried to fill out the informed consent form. The whole study procedure was approved by the Local Ethics Committee of Tabriz University of Medical Sciences (IR.TBZMED.VCR.REC.1398.025). All procedures were conducted according to the Declaration of Helsinki. Volunteers were requested to fulfill the consent form before getting involved in this research.

## Immunosensor assembly

### Electrode cleaning

Physical cleaning and electrochemical pre-treatment steps were performed before the biosensor fabrication. For the physical cleaning, GCE was polished with 0.3 and 0.05 µm alumina slurry on a polishing pad, followed by sonication in ethanol and then distilled water for 5 min, and finally dried with N_2_ gas flow. For electrochemical pre-treatment, GCE was rinsed with a 2% V/V nitric acid solution and then soaked in 0.5 M H_2_SO_4_, and the CV technique was run between − 0.3 and 1.55 V with a scan rate of 100mV/s until achieving repetitive cycles.

### Pt@ZIF-8 electrodeposition on GCE surface

The GCE was set in the cell containing 10 mL of Pt@ZIF-8, and afterward, the CV technique with 20 consecutive cycles in the potential range of − 0.4 to 1.44 V versus Ag/AgCl with a scan rate of 50 mV/s was conducted for electrochemical deposition of Pt@ZIF-8 on the electrode surface. The prepared Pt@ZIF-8/GCE was put in a PBS solution for 2 min and dried at room temperature. To the best of our knowledge, the Pt@ZIF-8 electrodeposition process on the GCE surface has not already been reported.

### Antibody immobilization

After electrodeposition of Pt@ZIF-8 on GCE, the electrode surface was prepared for antibody immobilization by incubating 10 µL of EDC/NHS aqueous solution on the electrode surface for 2 h at room temperature and then rinsing with PBS. Consequently, 10 µL of antibody at a concentration of micro-molar was cast on the Pt@ZIF-8/GCE surface and incubated for 2 h at room temperature, then rinsed with PBS buffer. 20 µL of 0.5% v/w BSA was then dropped on the anti-tau/Pt@ZIF-8/GCE surface and incubated for 1h at room temperature to barricade non-specific bindings.

### Electrochemical assay of *cis*-tau

To evaluate the analytical performance of the sensing interface, 10 µL of the *cis*-tau solutions in various concentrations were incubated on the BSA/Anti-tau/ Pt@ZIF-8/GCE surface for 1h at 25 °C. Then, the biosensor was cleansed with distilled water and put in the electrochemical cell containing 10 mL of 5 mM K_3_/K_4_[Fe(CN)_6_] and 0.1 M KCl solution. Thereupon, DPV techniques in the potential range of 0–0.5 V with a scan rate of 100 mV/s were carried out. The alteration in the peak currents (Ip) was relative to the concentrations of *cis*-tau protein captured on the immunosensor. Each step was repeated at least 3 times.

## Results and discussion

### The design of immunosensor

**Figure 1:**
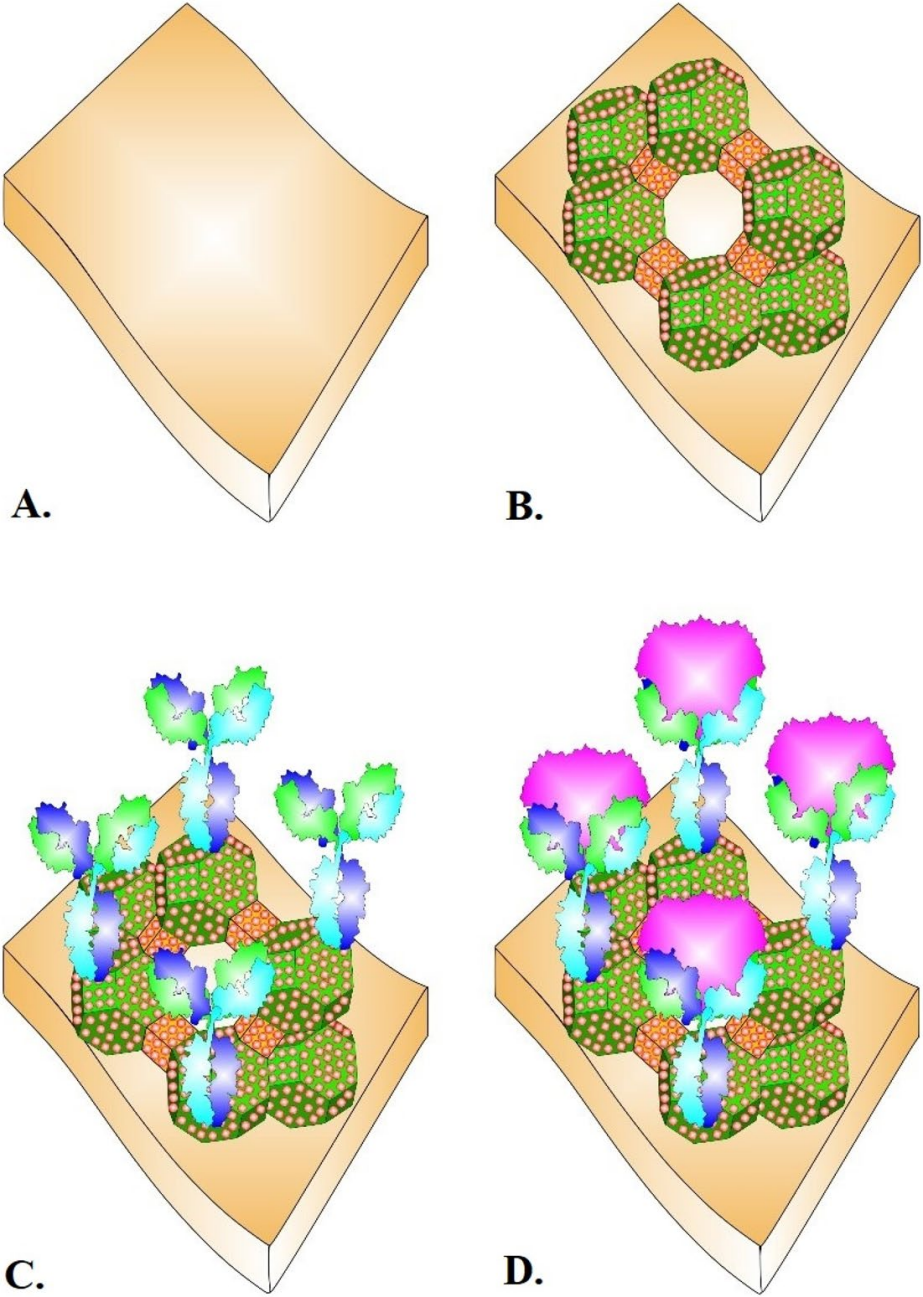
Schematic representation of immunosensor fabrication process. (**A**) Bare GCE; (**B**) Electrodeposited Pt@ZIF-8 on GCE; (**C**) Ab cis-p-tau immobilization on the modified surface; (**D**) The Ab *cis*-p-tau/pt@ZIF-8/GCE interaction with target *cis*-p-tau.

We developed a nanomaterial-based electrochemical immunosensor employing Pt@ZIF-8 and an antibody to boost electrochemical performance for *cis*-tau measurement in standard and clinical samples. Figure 1 shows the fabrication steps of the biosensor. Briefly, we synthesized colloidal Pt NPs and used them to prepare Pt@ZIF-8 nanocomposite through a one-step mechanism. Then, the nanocomposite was electrochemically deposited on the bare GCE surface. The COOH functional groups of the nanocomposite were activated by incubating EDC/NHS solutions on Pt@ZIF-8/GCE. Specific Ab of *cis*-tau was immobilized on the sensor surface, and BSA was used as a backfill agent to block Ab-free surface areas. Finally, various concentrations of *cis*-tau protein were incubated on the BSA/Anti-tau/Pt@ZIF-8/GCE surface. The functioning principle of this immunosensor is based on the change in charge transfer and impedance of the sensing platform caused by the binding of *cis-*tau protein to the immobilized Anti-tau receptors.

### Pt@ZIF-8 nanocomposite synthesis and characterization

The nature of the surface functional groups of Pd@ZIF-8 nanocomposite is investigated by the FT-IR technique. As is obvious in the FT-IR spectrum (Fig. S1), bands at 3100 and 1500 are attributed to the aromatic C–H and C–N stretch of the imidazole ring, respectively. Also, bands in the spectral regions below 800 cm^−1^ and 900–1350 cm^−1^ are respectively assigned to the out-of-plane and in-plane bending of the imidazole ring.

After the electrodeposition of Pt@ZIF-8 on the bare GCE surface, an SEM image was taken to investigate the morphology of the modified electrode surface and Pt NPs distribution. The FE-SEM images of the modified electrode are illustrated in Fig. 4A–C at different magnitudes.

**Figure 2:**
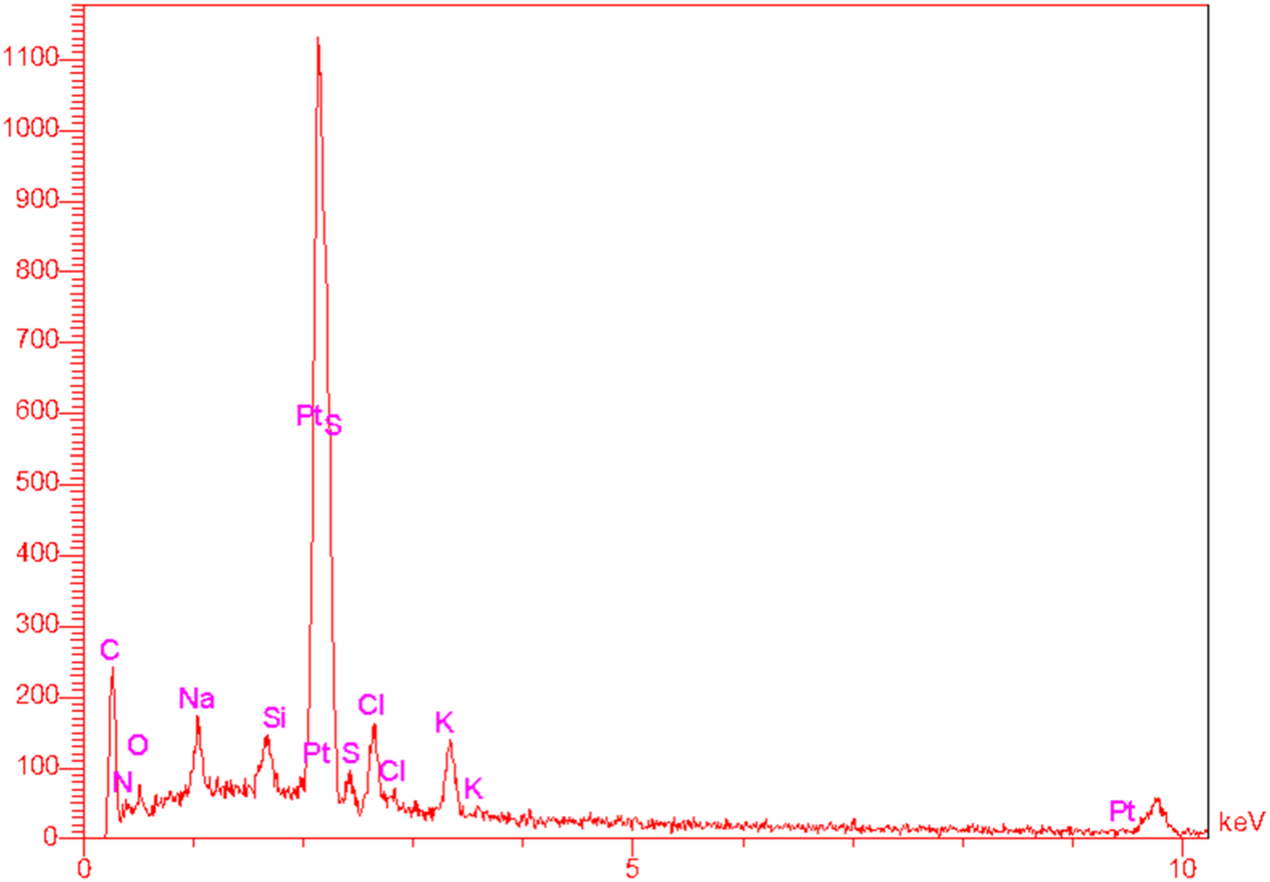
EDX analysis of Pt@ZIF-8/GCE surface.

As depicted in Fig. 4A–C, the surface morphology of the Pt@ZIF-8/GCE demonstrates several trunk-like projections posited on the whole surface. The Pt NPs with an approximate size ranging from 54 to 100 nm are distributed in the nanocomposite structure. For more confirmation, EDX was carried out to measure the chemical composition of Pt@ZIF-8. The EDX result presented in Fig. 2 confirms the existence of Pt (8.89%), C (34.06%), and N (19.68%) elements in nanocomposite structures. No impurities were detected in the nanocomposite structure.

SEM images and EDX analysis results infer the successful synthesis of the nanocomposite and its effectual deposition on the GCE surface.

We synthesized NPs of Pt noble metal in basic conditions through the ethylene glycol (EG)-based thermal reduction method^56^ that proceeds toward the surfactant-free preparation of Pt NPs colloid with confined particle size distribution. In this method, no surfactant like PVP was utilized to obtain stable colloids with small particles. This is the preferred advantage of this synthesis method compared to others^57^. It is assumed that the Pt NPs generation process could be described using the LaMer model^58^ in the following steps: monomer formation, nucleation, and growth. H_2_PtCl_6_·6H_2_O, EG, and sodium acetate were used as metal precursors, solvents, reducing agents, and stabilizers, respectively. While the metal precursor is reduced to provide Pt, EG is oxidized through various oxidation pathways to glycolaldehyde and glycol acid or to oxalaldehyde and oxalic acid. Owing to the presence of CH_3_COONa in the reaction mixture, CH_3_COO^−^ is crucial to stabilizing the produced Pt NPs as colloids. According to the LaMer model, after reducing metal precursor to form Pt monomers in supersaturated concentration, small nuclei are constructed. At the beginning of nucleation, a high concentration of CH_3_COO^−^ and just a couple of monomers are available in the reaction medium. This leads to monomer stabilization by CH_3_COO^−^ coordination. With further increments in Pt monomer concentration to a critical level, the small, unstable nuclei get sizeable enough to stay stable. The nucleation step continues until the Pt monomer concentration collapses below the nucleation threshold. In such a condition, the nucleation pathway stops and the particle growth step, as a competitive pathway, commences by depositing Pt monomers on the stable Pt nuclei.

According to results reported by Quinson et al. and Li et al. during surfactant-free synthesis of Pt NPs, CH_3_COO^−^ prevents not only NPs precipitation but also its enlargement during the growth step^54,59^. Thus, the diffusion-controlled growth step does not determine NPs size. In other words, CH_3_COO^−^ stabilizes the colloid and controls the NP size. Hence, It is highly probable that CH_3_COO^−^ anions get partially substituted by Cl^−^ halide ligands, leading to the halide-acetate mixed platinum complex [PtCl_x_(CH_3_COO^−^)_y_]^z-^ as an active intermediate species.

In continuation, the as-prepared colloidal Pt NPs were utilized to functionalize the ZIF-8 host matrix to obtain a heterogeneous nanocomposite with considerable physiochemical properties. ZIFs are a class of microporous materials with tetrahedral topologies^60^. They are generally constructed by kinking four-coordinated metals like Zn^2+^, Co^3+^, Cu^2+^, Ni^2+^, etc. through imidazole ligands. ZIF-8 was synthesized by continuous coordination of Zn^2+^ as the metal center and 2-methyl imidazolate units as the organic ligand^61^. The coordination of Zn^2+^ metal nodes with the nitrogen atoms at the 1, 3-positions of the organic ligands forms an extended framework of metal–imidazole–metal (M–Im–M).

Introducing pre-synthesized colloidal Pt NPs to the ZIF-8 synthesis procedure leads to Pt NPs encapsulation by ZIF-8. It means that guest species of Pt NPs are not embedded in the ZIF-8 pores. Rather, they are enclosed by the grown ZIF-8 frameworks to form core–shell heterostructures. It is probably because of the larger size of Pt NPs (about 95 nm observed in SEM images) compared with narrow cavity size distributions of 1–2 nm^54^. CH_3_COO^-^ as a capping agent grants a relatively large hydrodynamic radius to the Pt NPs which is the main determining reason for its size. Because of the mild synthesis condition, the structure of Pt NPs was well preserved. It is worth mentioning that the ZIF-8 structure holds a quantum confinement effect that barricades the aggregation of Pt NPs in the nanocomposite structure and retains its activity and stability.

### Electrochemical characterization of electrode surface modification

CV, DPV, and SWV measurements were carried out in 10 mL of 5 mM K_3_/K_4_[Fe(CN)_6_] and 0.1 M KCl solution to evaluate the fabrication process of the immunosensor either by approving the assembly and binding process or investigating the modified surface features as mentioned in

#### Fabrication of the immunosensor

The Pt@ZIF-8 nanocomposite was electrochemically coated on the GCE surface applying the CV technique. Figure S2 perspicuously shows the cyclic voltammogram recorded during the electrochemical deposition of Pt@ZIF-8 nanocomposite on a bare GCE surface. The distinguished peak in Fig. S2 is an indicator of nanocomposite oxidation on the bare GCE surface. A decrease in peak current is a prominent sign of successful deposition and changes in the surface after each cycle. Moreover, the oxidation peak of the first cycle is observed at about 0.8 V against the Ag/AgCl reference electrode. However, they shift towards larger potentials with repeated screening cycles.

It seems that Pt@ZIF-8 electropolymerization takes place via a radical chain reaction. The mechanism starts with anodic oxidation of the organic segment of Pt@ZIF-8 leading to emerging radical cation species. The coupling of the two radical cations produces a dimer specious. The electropolymerization proceeds by further oxidation of the dimer and its combination with another cation radical monomer. The polymer grows on the GCE surface through a continuous combination of monomer and oligomer species.

GCE modification by Pt@ZIF-8 leads to a considerable increase in redox peak current in cyclic, differential, and square pulse voltammograms compared with bare GCE, suggesting that Pt@ZIF-8 nanocomposite could outstandingly improve the electrochemical performance of bare GCE. This is mainly because of the synergetic effect between the great electrical conductivity of Pt NPs and the ordered crystalline pores of ZIF-8. Bin et al.^62^ reported that ZIF-8 is an inert compound and cannot solely enhance the electron transport rate at the GCE interface. Nevertheless, ZIF-8, with its high porosity and large surface area, provides the opportunity for high-density and monotonous encapsulation of Pt NPs to enhance conductivity.

Pt@ZIF-8/GCE contains a large number of COOH functional groups, proposing covalent immobilization of biorecognition elements as a beneficial option. With the intention of Ab covalent binding to the modified electrode surface, an aqueous solution of EDC/NHS was incubated on Pt@ZIF-8/GCE to obtain a surface layer containing succinimidyl ester (–COOSuc) terminals. Then, the surface was activated and ready for linkage to the amine (–NH_2_) groups of Anti-tau.

**Figure 3:**
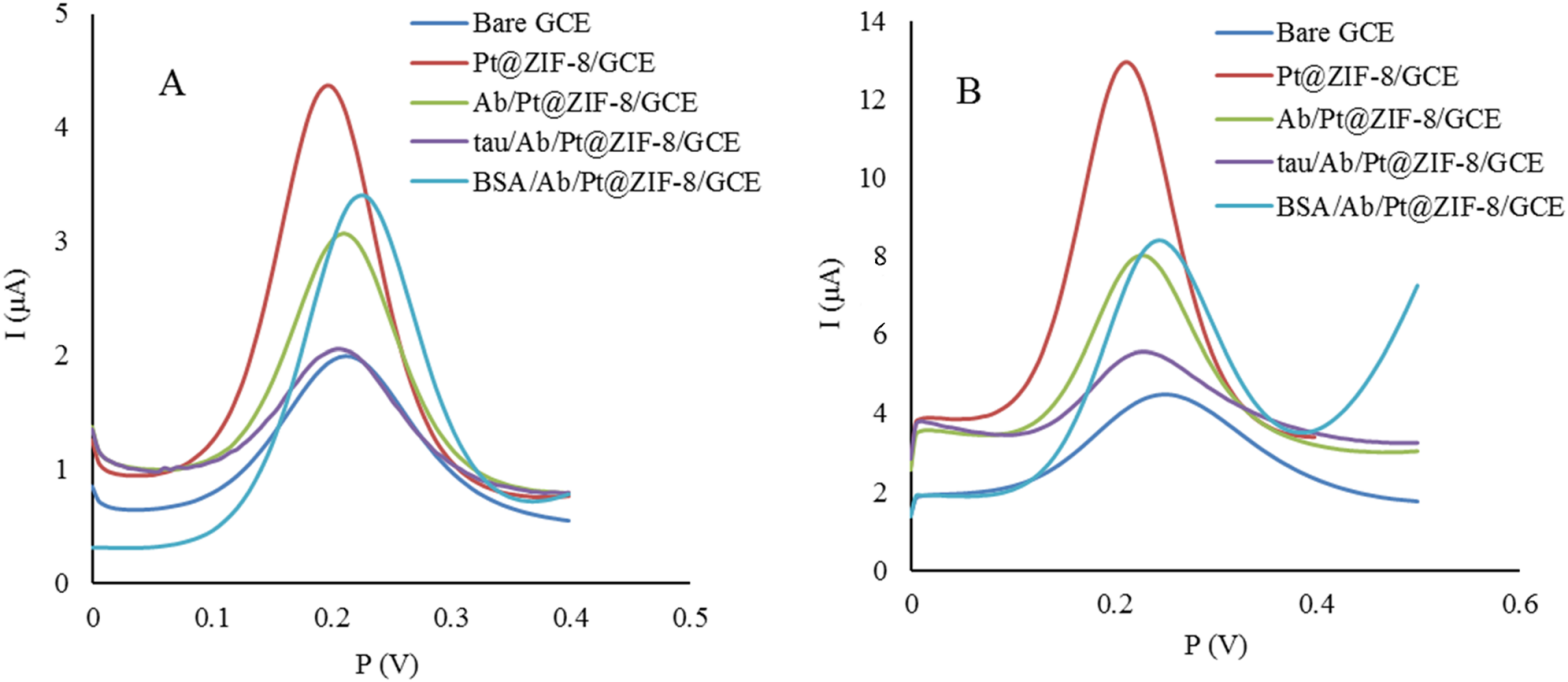
Evaluation of biosensor fabrication steps. (**A**) the DPV voltammograms (**B**) the SWV voltammograms. The electrochemical measurements were obtained in 5 mM K_3_/K_4_[Fe(CN)_6_] and 0.1 M KCl.

Anti-tau insertion on the electrode surface, through covalent NH-COO binding, led to a substantial decrease in peak current that is attributed to the formation of a shielding blocking layer via Anti-tau immobilization and its strong resistance to charge transfer across the sensor/electrolyte interface. It is noteworthy that Pt@ZIF-8 nanocomposite with a large surface area resulted in high Anti-tau dense loading on the modified surface and a notable decrease in electron transfer rate. Ab occupies active sites on the sensing surface and obstructs charge transfer with an electrolyte solution. In the next step, BSA was applied to pave the path towards the elimination of non-specifically absorbed Anti-tau and also the efficient orientation of the immobilized ones on the sensing platform. However, as observed in Fig. 3A,B, blocking the electrode surface with BSA had almost no effect on Ip. It means that Ab immobilization and coverage on the electrode surface were completely successful. Finally, the incubation with 10^−9^ µM *cis*-tau resulted in Ab-*cis*-tau complex formation on the modified GCE surface and Ip reduction in CV and DPV. The obtained results imply successful coverage of the GCE surface with Pt@ZIF-8, Ab covalent immobilization, and Ab-*cis*-tau formation.

As shown in Fig. 3, changes in the current intensity (Ip) of well-defined redox peaks are observed during surface modification. In the case of bare GCE, the potential difference (ΔEp) and the currents of the redox peaks in the cyclic voltammogram indicated a quasi-reversible redox reaction.

**Figure 4:**
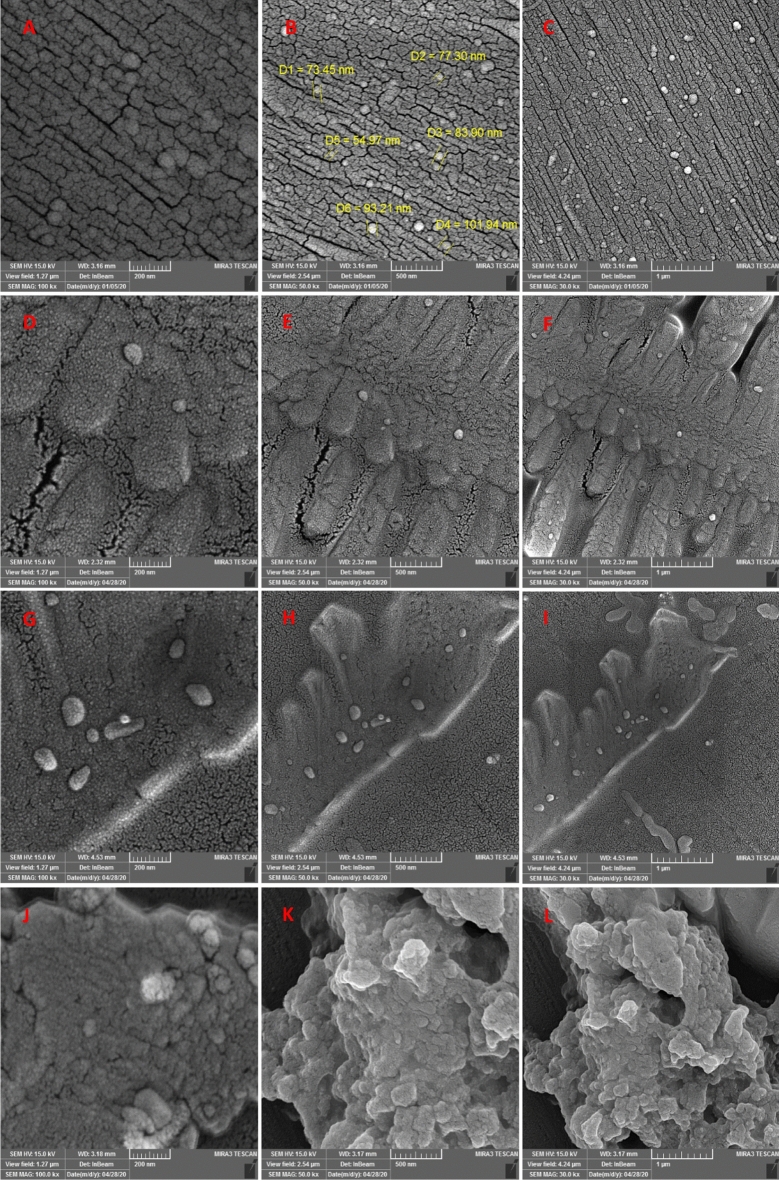
FE-SEM images of (**A**, **B**, and **C**) Pt@ZIF/ GCE; (D, E, and F) NHS/Pt@ZIF/GCE; (**G**, **H**, and **I**) Ab/EDC/NHS/Pt@ZIF/GCE; (**J**, **K**, and **L**) tau/Ab/EDC/NHS/Pt@ZIF/GCE.

FE-SEM images were recorded after each fabrication step of the immunosensor. Figure 4A–L depicts the surface morphology of the sensing platform after each preparation step. SEM technique was used to visualize the overall surface changes induced by the modifications. Changes in surface morphology are an indication of successful modification. As mentioned before, the surface morphology of the Pt@ZIF-8/GCE (Fig. 4A–C) demonstrates many trunk-like projections posited on the whole surface. Pt NPs with an approximate size ranging from 54 to 100 nm are distributed in the nanocomposite structure. Images 4D-F show the surface changes upon EDC/NHS addition. SEM is a useful technique for surface imaging, but it may not immediately reveal changes brought on by the addition of EDC/NHS or other chemical agents at the nanostructure level. Other microscopy methods like TEM and AFM with better resolution and sensitivity are more appropriate for a thorough investigation of such alterations. Antibodies are immobilized by being affixed to a surface or substrate. This process typically occurs at the nanoscale level and involves the interaction between molecules, which might not be easily visible with SEM. Furthermore, because antibodies are tiny molecules (around 10–15 nm in size), any alterations brought on by immobilization could be indistinguishable from conventional SEM resolution. When antibodies are immobilized on a substrate or nanomaterial, they can create a layer or pattern that may be visible in SEM images. SEM could be able to detect changes, such as the creation of clusters or aggregation of antibodies on the surface, as a result of the immobilization process. SEM can also show changes in the surface's general roughness or texture if the immobilization produces such changes. As observed in Fig. 4, no obvious difference in surface morphology occurred, which proves the monotonous deposition of the nanocomposite on the electrode surface and subsequently the uniform immobilization of the antibody on the surface without any aggregation. It's crucial to remember that SEM only reveals data about the general surface characteristics; it cannot directly see specific antibody molecules or the specifics of their interactions with the substrate. More specialized methods, like TEM or AFM, would be more suitable for that degree of examination. Finally, images 4J-L depict the localization of tau protein on the sensing platform.

### Analytical approach

**Figure 5:**
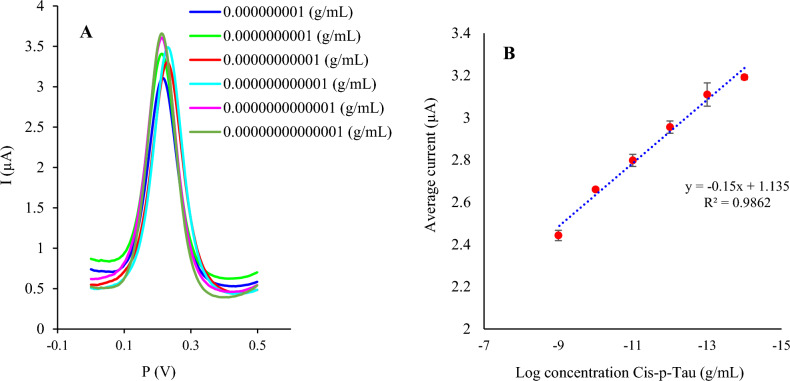
The analytical efficiency of the assembled immunosensor in a concentration range of 10 ng/mL–1 fg/mL of *cis*-tau. (**A**) DPVs and (**B**) the calibration curve of the obtained signals for different concentrations of *cis*-tau (n = 3). The electrochemical measurements were conducted in 5 mM K_3_/K_4_[Fe(CN)_6_] and 0.1 M KCl.

To verify the analytical performance of the developed immunosensor, DPVs were recorded by incubating different concentrations of cis-tau (10 ng/mL to 1 fg/mL) on the biosensor. According to the results, Ip values decrease after Ab-*cis*-tau complex formation since *cis*-tau binding to the Ab inhibits electron transfer across the biosensor/electrolyte interface. As indicated in Fig. 5, the calibration plot displays a desired linear relationship in the 10 ng/mL to 1 fg/mL concentration range between the peak currents and Log (concentration). The regression equation is expressed as I = − 0.15 Log C + 1.135 (R^2^ = 0.9862). This method exhibits a low detection limit of 1 fg/mL.

### Method optimization

To improve the analytical performance of the fabricated immunosensor, experimental variables, including the number of Pt@ZIF-8 electrodeposition cycles, Anti-tau incubation time, and temperature, were optimized. The effect of each variable was examined in 10 mL of 5 mM K_3_/K_4_[Fe(CN)_6_] and 0.1 M KCl solution employing CV, DPV, and SWV techniques.

Figure S3 indicates the effect of Pt@ZIF-8 electrodeposition cycles’ number on DPV peak current. The CV technique in 5 to 30 cycles was conducted for Pt@ZIF-8 electropolymerization on the GCE surface. Raising the number of cycles from 5 to 20, the oxidation peak current increased, and the highest peak current was obtained when 20 cycles of CV were conducted. When the number of cycles exceeded 20, the peak current deceased because of the excessive and hindering thickness of Pt@ZIF-8 nanocomposite, which clogged the electron transfer. Therefore, the Pt@ZIF-8 electrodeposition process was favorably completed in 20 cycles.

To find out the optimum Anti-tau incubation temperature on the Pt@ZIF-8/GCE surface, the incubation process at 4, 25, and 37 °C was examined. According to the reductions in peak currents of DPV indicated in Fig. S4, the anti-tau immobilization temperature of 25 °C caused the greatest dense loading of Anti-tau and consequently the greatest reduction in Ip. Probably at 4 and 37 °C, the molecular dynamics of Anti-tau do not favor its optimal orientation for efficient covalent binding.

Incubating anti-tau at 25 °C for 1 h and 2 h. Data revealed that 2 h was the appropriate incubation time. The 2 h incubation time provided the opportunity for immobilizing the maximum amount of Anti-tau and consequently the largest Ip reduction. CV, DPV, and SWV voltammograms shown in Fig. S5 approve 2 h as the optimum incubation time.

### Real sample analysis of cis-tau protein

To examine the clinical performance of the immunosensor for clinical bioanalysis, five serum samples from Alzheimer patients were analyzed. To achieve this aim, samples were obtained from Shahid Ghazi Hospital affiliated with Tabriz University of Medical Sciences. All patients were queried to fill out the informed consent form. The *cis*-tau protein level in the serum samples was measured by the designed immunosensor, indicating a correlation between the oxidation current response and, the *cis*-tau level. The results endorse the feasibility of the constructed immunosensor to sense low levels of *cis*-tau in human serum samples (Fig. S6).

Compared with former electrochemical methods listed in Table 2 for tau protein detection, the proposed immunosensor indicated significant improvements in tau detection, like a simple fabrication process, a wide detection range, and a low detection limit, as well as specificity for *cis*-tau detection in human serum samples. Since the enzyme-linked immunosorbent assay (ELISA) reference technique is not able to detect lower concentrations of cis-tau, it is not comparable to the developed *cis*-tau immunosensor.

**Table 2:** Electrochemical biosensors for tau protein detection.

Technique	Sensing platform	Sample	Linear range	LOD	Refs.
EIS	Ab/Protein G/3,3'-dithiobis(sulfosuccinimidyl propionate) (DTSSP)/Gold Electrode	BSA & Human serum	0.01 pM to 10 nM	0.03 pM	^63^
DPV	Aptamer-Au-CS bioconjugate /BSA/Ab/MPA/Gold Electrode	Human serum	0.5 pM to 100 pM	0.42 pM	^64^
EIS	3-aminophenol MIP/C-SPE	PBS	2.18 pM to 2.18 nM	0.024 pM	^65^
EIS	n-butylamine/Ab/lipoic acid/Au Electrode	PBS	–	100 μg/mL	^66^
DPV	carbon nanocomposite film composed of MWCNTs, rGO, and CS/Gold Electrode	Human serum	0.5–80 fM	0.46 fM	^67^
DPV	Apt/AuNPs/Paper-based vertical graphene electrode (VGE)	Blood	0.1 pg/mL to 1 ng/mL	0.034 pg/mL	^68^
SWV, CV	Ab/MWCNTs-PAH/Pt/C-SPE	Serum	8.6–1100 pg/mL	0.24 pg/mL	^69^
Amperometric	HRP-DAb/tau/capture antibody/AuNPs-PAMAM/C-SPE	Undiluted human plasma and brain tissue extracts	11–800 pg mL	1.7 pg mL–1	^70^
SWV	Ab/rGO/GCE	Serum	0.08 pM-80 pM	75 fM	^71^
DPV	/carboxyl graphene/thionin/gold NPs/GCE	Serum	1.0 pM to 100 pM	0.70 pM	^72^
EIS	Ab/Flower-shaped TiO2/Gold Electrode	Serum	1–200 ng/mL	1.774 pg/mL	^73^
DPV, EIS	BSA/Ab/Pt@ZIF-8/GCE	Serum	1 fg mL^−1^ to 10 ng mL^−1^	1 fg mL^−1^	This work

### Reproducibility, specificity, and stability of immunosensor

The reproducibility of the Anti-tau/Pt@ZIF-8/GCE was also examined by the inter-assay technique. Three independently prepared electrodes in equal condition through an identical assembly process were used to detect 10^−9^ µM of *cis-*tau by conducting the DPV technique. The relative standard deviation (RSD) was obtained at 9.11% (Fig. S7). This indicates adequate reproducibility of the immunosensor.

**Figure 6:**
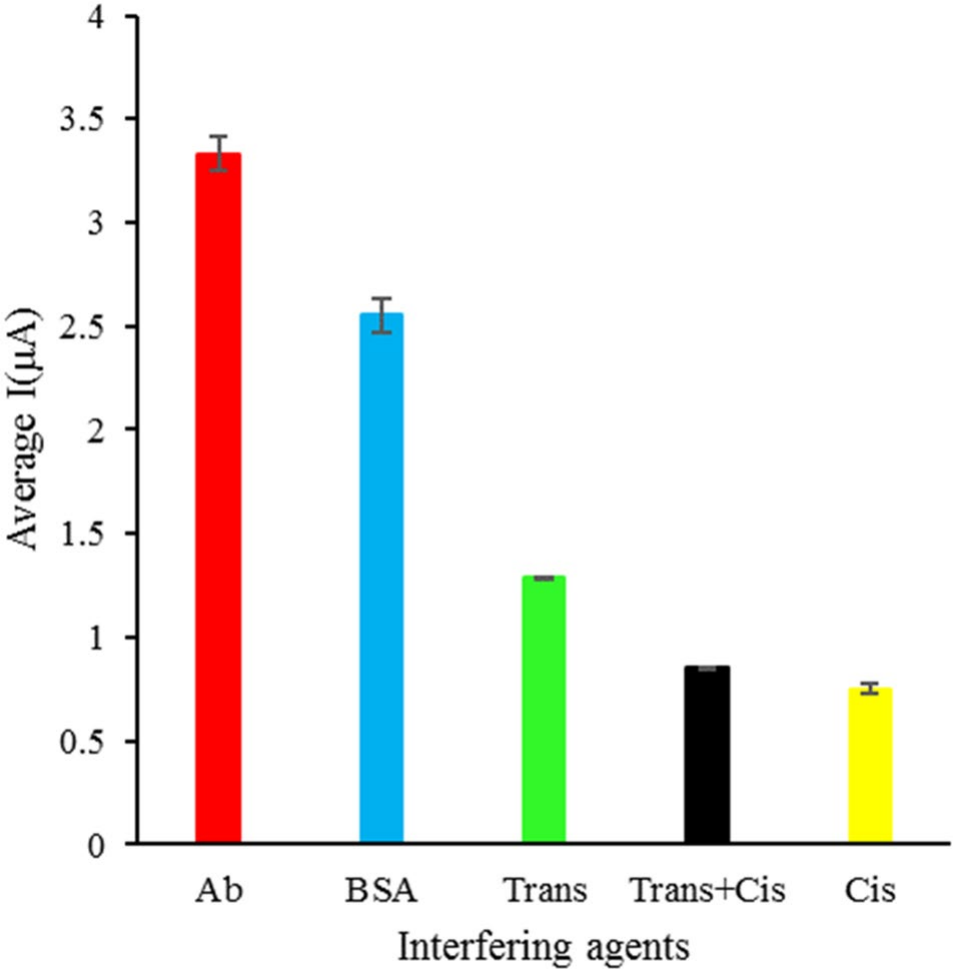
The selectivity of the immunosensor for cis-tau protein against three possibly interfering agents. The electrochemical measurements were conducted in 5 mM K3/K4[Fe(CN)6] and 0.1 M KCl.

To evaluate the specificity of the biosensor, DPV responses of the immunosensor to possible nonspecific interfering agents such as bovine serum albumin, trans-p-tau, and total tau were recorded in a 100-fold concentration of *cis*-tau. As can be seen in Fig. 6, the Ip of the target protein sample is stronger than that of other nonspecific interfering agents. This shows the acceptable specificity of the developed immunosensor.

Lastly, the inter-day stability of the designed immunosensor was also investigated to affirm the precision of the proposed biosensor, especially for practical applications. For the inter-day stability investigation, BSA/Anti-tau/Pt@ZIF-8/GCE was prepared and stored at 4 °C for 7 days. The DPV technique was conducted for 7 consecutive days at 24-h intervals. According to the differential pulse voltammograms presented in Fig. S8, no obvious change in Ip was recorded, and 85% of its initial response was retained after 7 days.

## Conclusion

In summary, a new label-free electrochemical immunosensor is reported for selective and sensitive detection of *cis*-tau as an AD biomarker. Colloidal Pt NPs were synthesized through the ethylene glycol (EG)-based thermal reduction method. Then, Pt NPs were employed for Pt@ZIF-8 nanocomposite synthesis. The nanocomposite was electrochemically coated on the GCE surface applying the CV technique. The synergetic effect between the great electrical conductivity of Pt NPs and the ordered crystalline pores of ZIF-8 enhances the efficient surface area and conductivity of GCE. The EDC/NHS covalent coupling system was utilized for surface activation before Anti-tau immobilization. By immobilizing Anti-tau on Pt@ZIF-8/GCE, the biosensor assembly process is completed. Morphological and electrochemical analyses were performed to prove the successful fabrication of the immunosensor. The results indicate acceptable specificity, reproducibility, and stability of the sensing platform. Under the optimized experimental conditions, the proposed immunosensor exhibited strong analytical performance for cis-tau detection in standard and human serum samples in a linear range of 1 fg mL^−1^ to 10 ng mL^−1^ with a low detection limit of 1 fg mL^−1^. The ELISA reference method is not able to measure pico- or femtomolar concentrations of *cis*-tau; therefore, it is not comparable to the designed *cis*-tau immunosensor.

### Supplementary Information

Supplementary Figures.

## Data Availability

All data generated or analyzed during this study are included in this published article.
