# Supplementary material for: Application of Pt@ZIF-8 nanocomposite-based electrochemical biosensor for sensitive diagnosis of tau protein in Alzheimer’s disease patients
Source: Sci Rep. 2023 Sep 27;13:16163. doi: 10.1038/s41598-023-43180-0 (PMC10533502; doi:10.1038/s41598-023-43180-0)
Supplement: Supplementary file 1 — Supplementary Figures. [file 41598_2023_43180_MOESM1_ESM.docx]

**Application of Pt@ZIF-8 nanocomposite-based electrochemical biosensor for sensitive diagnosis of tau protein in Alzheimer’s disease patients**

Forough Chakari-Khiavi^1,2^, Arezoo Mirzaie^3^, Balal Khalilzadeh^4, 5*^, Hadi Yousefi^6^, Rozita Abolhasan^7^, Amin Kamrani^7^, Ramin Pourakbari^7^, Koorosh Shahpasand^8^, Mehdi Yousefi^4^, Mohammad-Reza Rashidi^9,*^

1. Department of medicinal chemistry, Faculty of Pharmacy, Tabriz University of Medical Sciences, Tabriz, Iran
2. Student research committee, Tabriz University of Medical Sciences, Tabriz, Iran
3. Pharmaceutical Analysis Research Center, Tabriz University of Medical Sciences, Tabriz, Iran
4. Stem Cell Research Center (SCRC), Tabriz University of Medical Sciences, Tabriz, Iran
5. Hematology and Oncology Research Center, Tabriz University of Medical Sciences, Tabriz, Iran
6. Department of Basic Medical Sciences, Khoy University of Medical Sciences, Khoy, Iran
7. Department of Immunology, Faculty of Medical Sciences, Tabriz University of Medical Sciences, Tabriz, Iran
8. Department of Stem Cells and Developmental Biology, Cell Science Research Center, Royan Institute for Stem Cell Biology and Technology, Academic Center for Education, Culture and Research (ACECR), Tehran 1665659911, Iran
9. Research Center for Pharmaceutical Nanotechnology (RCPN), Tabriz University of Medical Sciences, Tabriz, Iran

**Corresponding authors**

***Balal Khalilzadeh****, Ph.D, Stem Cell Research Center (SCRC), Tabriz University of Medical Sciences, 51664-14766 Tabriz- Iran, Tel: +98(41)-33363311; Fax: +98(41)33363231.*

*Email:* [*balalkhalilzadeh@gmail.com*](mailto:balalkhalilzadeh@gmail.com)*, khalilzadehb@tbzmed.ac.ir*

***Mohammad-Reza Rashidi****, Ph.D, Department of Department of Medicinal chemistry, Faculty of pharmacy, Tabriz University of Medical Sciences, Tabriz, Iran 
PO Box: 6446-14155, Email:* [*rashidi@tbzmed.ac.ir*](mailto:rashidi@tbzmed.ac.ir)

**Figure S1**


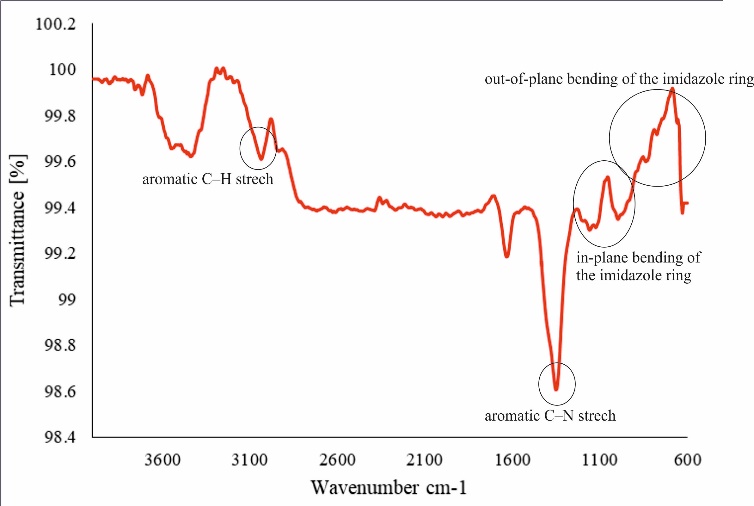


Figure S1. FT-IR spectrum of Pt@ZIF-8

**Figure S2**

Figure S2. Cyclic voltammogram of Pt@ZIF-8 nanocomposite electrodeposition on GCE surface through

20 successive cycles in the potential range of -0.4 to +1.4 V.

**Figure S3**


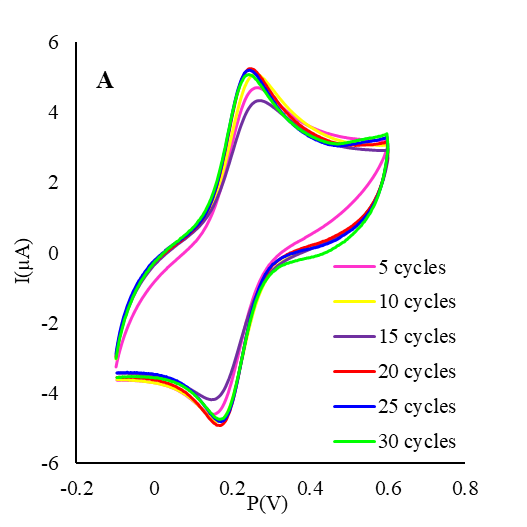

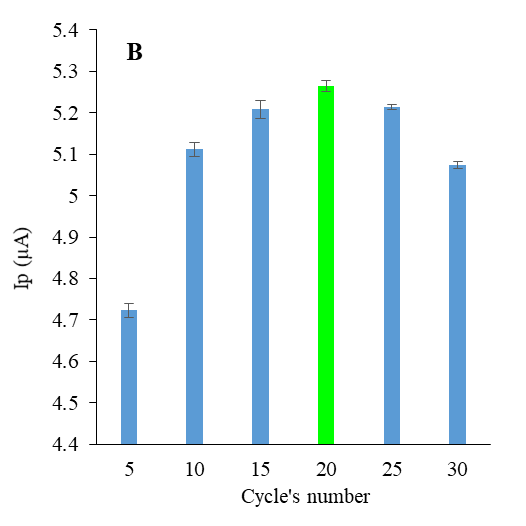

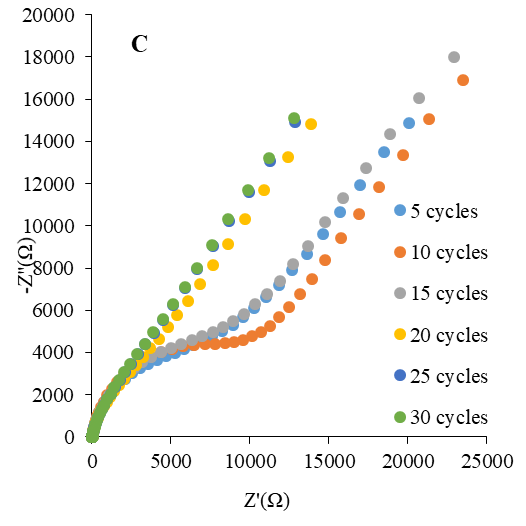


Figure S3. The number of electrodeposition cycles effect (A) Cyclic voltammograms (B) Histogram of the Ip (C) EIS plot. The electrochemical measurements were conducted in 5 mM K_3_/K_4_[Fe(CN)_6_] and 0.1 M KCl.

**Figure S4**


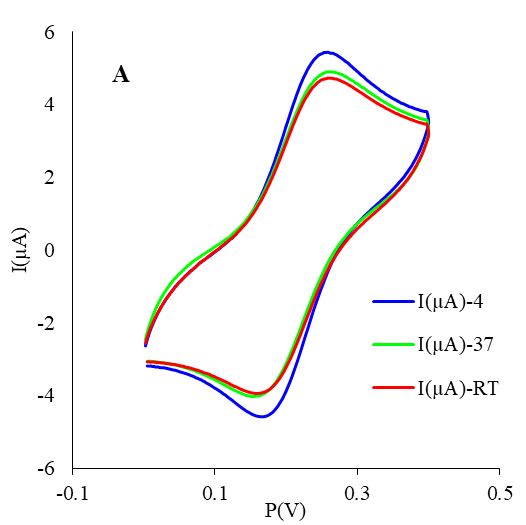

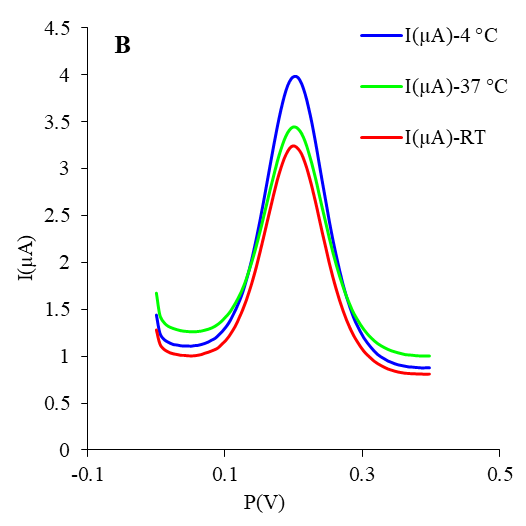

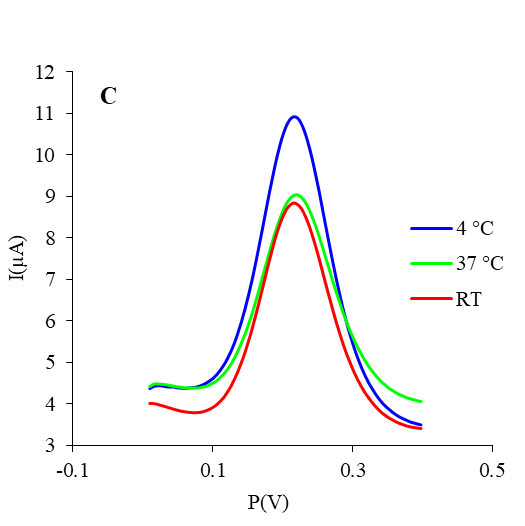

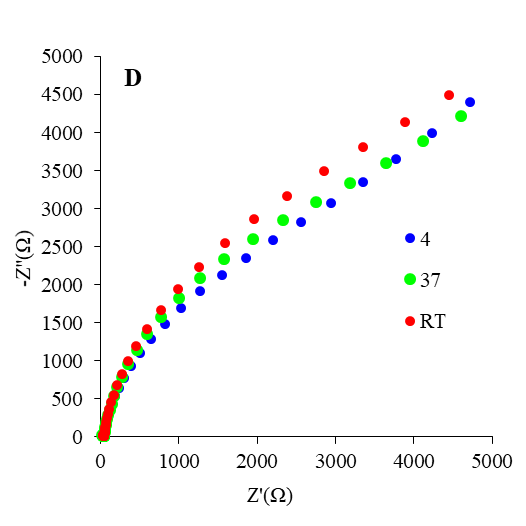


Figure S4. Anti-tau incubation temperature optimization (A) CVs (B) DPVs (C) SWVs (D) EIS plots. The electrochemical measurements were conducted in 5 mM K_3_/K_4_[Fe(CN)_6_] and 0.1 M KCl.

**Figure S5**


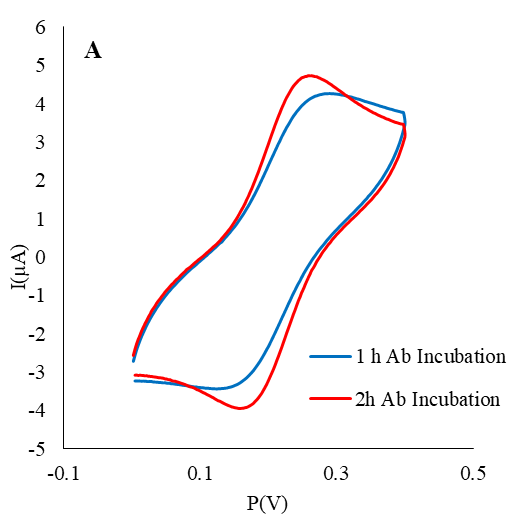

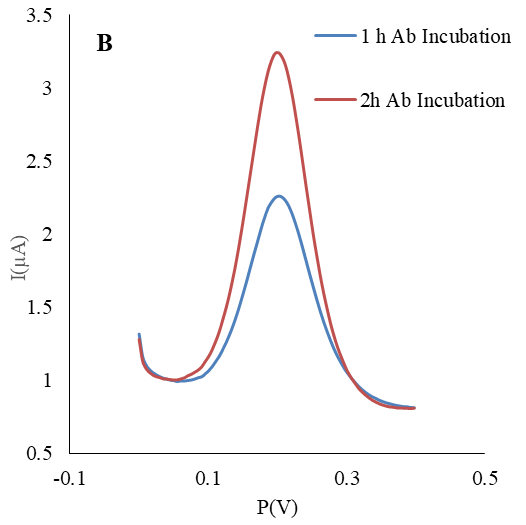

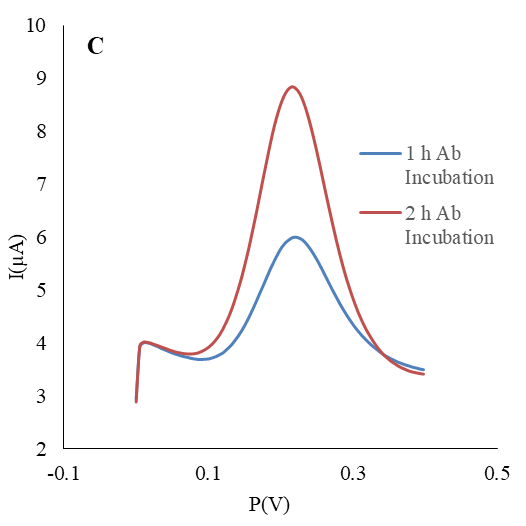


Figure S5. Anti-tau incubation time optimization (A) CVs (B) DPVs (C) SWVs. The electrochemical measurements were conducted in 5 mM K_3_/K_4_[Fe(CN)_6_] and 0.1 M KCl.

**Figure S6**

Figure S6. Analysis of real samples of AD positive patients. The electrochemical measurements were conducted in 5 mM K_3_/K_4_[Fe(CN)_6_] and 0.1 M KCl.

**Figure S7**

Figure S7. The signal reproducibility of the proposed biosensor. The electrochemical measurements were conducted in 5 mM K_3_/K_4_[Fe(CN)_6_] and 0.1 M KCl.

**Figure S8**


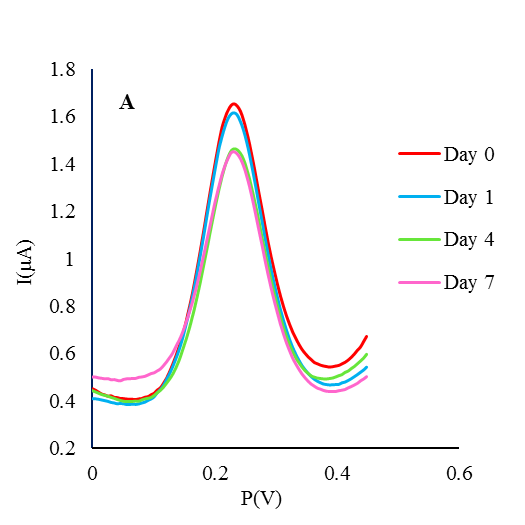

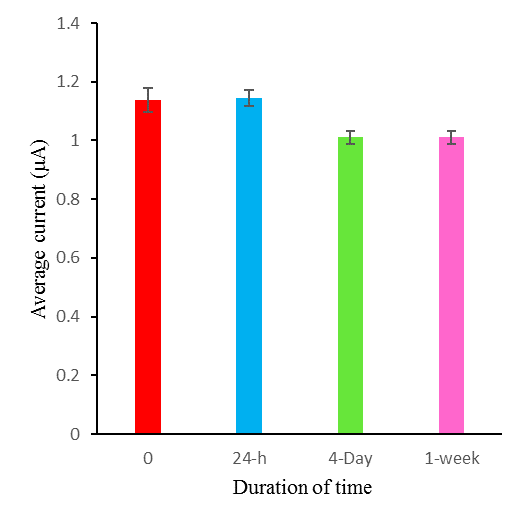


B

Figure S8. Inter-day stability of the proposed sensing platform (A) DPVs (B) histograms. The electrochemical measurements were conducted in 5 mM K_3_/K_4_[Fe(CN)_6_] and 0.1 M KCl.
